# Rare Case of Botulism and Dysphagia: The Role of Physical and Rehabilitation Medicine

**DOI:** 10.7759/cureus.48964

**Published:** 2023-11-17

**Authors:** André Teixeira, Nuno Lopes, Ana Pereira, Marta Amaral-Silva, Ana Catarina Miguéns

**Affiliations:** 1 Physical Medicine and Rehabilitation, Centro Hospitalar Universitário de Lisboa Central, Lisbon, PRT

**Keywords:** foodborne botulism, peripheral facial palsy, acute flaccid paralysis, neurological rehabilitation, oropharyngeal dysphagia

## Abstract

Botulism is a rare cause of flaccid paralysis resulting from the neurotoxin produced by the bacterium *Clostridium botulinum*. It is clinically characterized by symmetric proximal-distal paralysis, diplopia, dysarthria, dysphonia, and dysphagia. Definitive diagnosis requires laboratory confirmation through the detection of the toxin in blood, vomit, or stool samples. Treatment with antitoxin should be promptly initiated upon clinical suspicion and in the presence of epidemiological support due to evidence of reduced mortality. Physical and rehabilitation medicine plays a fundamental role in the recovery of deficits and prevention of complications. In this report, the authors describe a 19-year-old patient with botulism with dysphagia, dysphonia, and facial paresis in the rehabilitation ward.

## Introduction

Botulism is a rare and potentially fatal condition characterized by symmetrical proximal-to-distal flaccid paralysis resulting from exposure to the neurotoxin produced by the bacterium Clostridium botulinum. There are eight toxin types (A-H), with types A, B, E, and F being the most toxic to humans. The toxin reaches nerve endings through the circulatory system and can bind to pre-synaptic receptors in both motor and autonomic nerves. It is internalized within the nerve cell and inhibits the release of acetylcholine in the synaptic cleft, leading to a blockade of cholinergic neuromuscular activity in smooth and striated muscles and sweat, salivary, and lacrimal glands [1]. The bacterium can be found on the surface of foods, in soil, and in aquatic environments, thriving particularly in anaerobic conditions [2]. Botulism presents in various forms: infantile, foodborne, wound-associated, intestinal toxemia, and bioterrorism-related. Clinical features primarily involve cranial nerves, resulting in diplopia, dysarthria, dysphonia, and dysphagia, typically appearing 12 to 72 hours post-exposure [1-3]. The absence of fever or sensory deficits, normal mental state, and hemodynamic stability are also characteristic features [4]. Definitive diagnosis is established through the isolation of the toxin in ingested food, blood, vomit, or stool. However, in cases of clinical suspicion, prompt initiation of antitoxin therapy is crucial [4-5]. This article describes a case of botulism with a focus on the role of physical and rehabilitation medicine (PRM) intervention.

## Case presentation

A previously healthy 19-year-old university student, with a two-week history of dry cough presented to the emergency department with the sudden onset of dysphonia and decreased tongue strength. Initially, he was discharged with a referral to the otorhinolaryngology department. However, the following day, he developed facial muscle weakness and had difficulty swallowing liquids, which prompted his return to the emergency department. He reported recent changes in bowel movements, including a single episode of liquid stool without blood, mucus, or pus. Due to the suspicion of Miller Fisher variant Guillain-Barré syndrome, he was transferred to a specialized referral hospital. Upon admission, he exhibited blurred vision, bilateral peripheral facial palsy (House-Brackmann Grade 3), dysarthria, dysphonia, weak soft palate elevation, tongue protrusion with reduced muscular strength, and mixed dysphagia. His deep tendon reflexes were symmetric and hyperkinetic, with bilateral Hoffmann signs. Intravenous human immunoglobulin therapy was initiated, and he was transferred to the intensive care unit due to the risk of respiratory compromise. Considering the clinical presentation and epidemiological history of consuming canned food of dubious preservation in the two weeks preceding symptom onset, a presumptive diagnosis of foodborne botulism was made (Table 1). This diagnosis was later confirmed by a positive enzyme-linked immunosorbent assay (ELISA) test in the blood. Anti-toxin treatment was initiated on day 10 of symptoms, leading to progressive improvement in deficits, particularly orofacial motility and dysphagia. He was then transferred to the PRM ward. Additional findings from complementary studies included serology panels showing recent infections with respiratory syncytial virus and adenovirus, and previous infections with echovirus, Coxsackie A7/A9/B1-B5, cytomegalovirus, Epstein Barr, Rickettsia conorii, Chlamydia trachomatis, and Chlamydia pneumonia; negative autoimmunity panel; normal cerebrospinal fluid; cervical computed tomography (CT) revealing adenopathy in the internal jugular and accessory spinal chains; chest CT and cranial magnetic resonance imaging without abnormalities. Upon transfer to the Physical and Rehabilitation Medicine (PRM) department, he continued to experience aggravated dysphonia with phonatory efforts, bilateral peripheral facial palsy (House-Brackmann Grade 2), altered tongue motility, reduced cough efficiency, and oropharyngeal dysphagia with rapid fatigue during swallowing. Functionally, he had modified independence, scoring 115/126 on the functional independence measure (FIM). Due to the swallowing impairments, he was maintained on enteral feeding via a nasogastric tube (NGT) and started an intensive rehabilitation program, including speech therapy and physiotherapy. An endoscopic assessment of swallowing on day 20 of the illness revealed inefficient swallowing and fatigue after brief periods, with residue persistence in the valleculae, piriform sinuses, and epiglottis. Despite the absence of clear signs indicating swallowing safety issues, the significant impairment in efficiency with rapid fatigue and residue persistence leads to continued NGT feeding with increased protein reinforcement and progressive swallowing training. During his hospitalization, there was progressive improvement in voice quality, swallowing efficiency, and tongue and facial motility (House-Brackmann Grade I). A repeat endoscopy of swallowing on day 44 showed good laryngeal mobility, improved oral sphincter tone with less fatigue during swallowing, and no signs of penetration or aspiration, allowing for NGT removal (summary of clinical and endoscopic evaluation of dysphagia in Table 2). Upon discharge, he was recovered and fully independent in activities of daily living with a FIM score of 126/126.

**Table 1:** Clinical criteria tool for early suspicion of botulism *The three clinical criteria must be present to suspect botulism. Originally published in Rao AK, Lin NH, Griese SE, Chatham-Stephens K, Badell ML, Sobel J. Clinical criteria to trigger suspicion for botulism: an evidence-based tool to facilitate timely recognition of suspected cases during sporadic events and outbreaks. Clin Infect Dis 2017;66(suppl_1):S38–S42 [6]. Used with copyright permission from the original publisher.

Clinical criteria*
1) Afebrile (≤38°C)
2) At least one of the following symptoms:
Blurred vision
Double vision
Difficulty speaking, including slurred speech
Any change in the voice, including hoarseness
Dysphagia/ pooling of secretions/ drooling
Thick tongue
3) At least one of the following signs:
Ptosis
Extraocular palsy or fatigability
Facial paresis
Fixed pupils
Descending paralysis, beginning with cranial nerves

**Table 2:** Clinical and endoscopic evaluation of dysphagia during hospitalization HB - House-Brackmann; N/A - Not Available

Patient evaluation	Specification of the evaluation	Day 10	Day 20	Day 44	Day 60
Clinical evaluation	Consciousness state/Orientation/Collaboration	Normal/ oriented/ cooperative.	Normal/oriented/cooperative.	Normal/ oriented/ cooperative.	Normal/ oriented/ cooperative.
	Verbal articulation	Mild dysarthria.	Slightly dysarthria.	Normal.	Normal.
	Voice quality	Hypernasality with changes in vocal quality.	Hypernasality with changes in vocal quality.	Mild hypernasality with evident improvement in vocal quality.	Almost normal.
	Facial muscle strength	Bilateral peripheral facial palsy (HB Grade 3)	Bilateral peripheral facial palsy (HB Grade 2)	Bilateral peripheral facial palsy (HB Grade 1).	Normal.
	Tongue strength and mobility	Protrusion in the midline, slowed lateral mobility without strength against resistance.	Protrusion in the midline, slowed lateral mobility with strength against mild resistance.	Protrusion in the midline with good mobility and strength against resistance.	Protrusion in the midline with normal mobility and strength.
	Cough efficacy	Weak voluntary cough capacity.	Weak voluntary cough capacity.	Effective voluntary cough capacity	Effective voluntary cough capacity.
	Gag reflex	Decreased.	Decreased.	Decreased.	Normal.
	Volume-viscosity swallow test	N/A	No signs of reduced safety were observed, but there was an important decrease in effectiveness with nectar and pudding consistency (rapid fatigue).	There were no signs of reduced safety. Substantial improvement in efficiency, able to swallow faster pudding consistency without fatigue.	Safety and effectiveness with all consistencies and volumes.
Flexible Endoscopic Evaluation of Swallowing	Non-food phase	N/A	Left nasopharynx permeable, free cavum, complete palate closure with [p], but incomplete with [t] and [k], the base of the tongue without alterations, no salivary stasis in any of the pharyngeal levels or over the glottis. Mobile vocal cords, with slight leakage during phonation. Non-functioning ventricular bands during phonation. Reduced vocal amplitude. Cough with reduced intensity. Laryngeal sensitivity preserved.	Compared to the previous evaluation, laryngeal sensitivity remains intact, and laryngeal mobility also shows good range with a cough of good intensity.	N/A
	Food phase	N/A	PUDDINGY: Functioning oral sphincter, but with a low tone. Correct intraoral retention. No spillage. Delay in laryngeal ascent during swallowing. Partially effective swallowing, with residues in the valleculae, pyriform sinus, and retrocricoid area, and not mobilizable after multiple swallows. After the 4th spoonful of porridge, residues on the laryngeal surface of the epiglottis. No penetration or aspiration was observed. LIQUID: Similar to the puddingy phase with no observation of penetration or aspiration.	PUDDINGY: Increased tone of the oral sphincter and improved swallowing efficiency with reduced retention in the valleculae and piriform sinuses. LIQUID: Similar to the puddingy phase.	N/A

## Discussion

Foodborne botulism, a notifiable disease, is a rare but potentially fatal condition that highlights the significance of early suspicion and diagnosis. The associated mortality rate has significantly decreased due to advancements in respiratory support care, as death can arise from respiratory failure or the repercussions of extended paralysis. The sudden onset of the clinical presentation, characterized by symmetric involvement of multiple cranial nerve pairs, preservation of sensitivity, deep tendon reflexes, consciousness, hemodynamic stability, and afebrile status, along with a favorable response to antitoxin strongly supports the diagnosis of botulism, which was later definitively confirmed by serum toxin detection [4,7,8]. While it would have been important to perform a microbiological analysis of the food to confirm the source of contamination, this was not possible. Nevertheless, differential diagnoses were ruled out through laboratory and radiologic tests, including variants of Guillain-Barré syndrome and myasthenia gravis. Early suspicion based on the clinical and epidemiological history played a crucial role in the prognosis, enabling the timely administration of antitoxin. Although antitoxin does not reverse established paralysis, it reduces mortality, with the greatest benefit when administered within the first 48 to 96 hours [8]. Dysphagia represents one of the most concerning manifestations due to the high morbidity and mortality risk associated with aspiration and pneumonia. In this context, repeated clinical and instrumented assessments of swallowing were crucial for the safe progression of the rehabilitation program. Furthermore, given the prolonged nature of recovery spanning weeks to months, the rehabilitation program played a crucial role in achieving a favorable outcome in this case. The program, tailored to enhance orofacial motility, voice retraining, and swallowing training, ultimately contributed to a complete recovery of deficits after a two-month hospitalization.

## Conclusions

In this rare case of botulism with dysphagia, the authors highlight the importance of early suspicion and diagnosis in the patient's outcome. Timely clinical suspicion and the administration of antitoxin therapy significantly contributed to a favorable deficit recovery. Dysphagia, a common and concerning symptom in botulism, was effectively managed through repeated assessments and a comprehensive rehabilitation program, emphasizing orofacial motility, voice, and swallowing retraining. This multimodal approach led to a complete recovery of deficits after a two-month hospitalization. In summary, this case emphasizes the crucial role of PRM in optimizing the recovery process for patients with botulism, particularly in addressing facial palsy and dysphagia, ultimately enabling a safe return to oral feeding.
